# A Rare Case of Hereditary Hemochromatosis Presenting With Porphyria Cutanea Tarda

**DOI:** 10.7759/cureus.41299

**Published:** 2023-07-03

**Authors:** Neilmegh Varada, Kyaw Min Tun, Mark J Chang, Shana Bomberger, Randy Calagari

**Affiliations:** 1 Internal Medicine, Kirk Kerkorian School of Medicine at the University of Nevada, Las Vegas, Las Vegas, USA; 2 Primary Care, VA Southern Nevada Healthcare System, Las Vegas, USA; 3 Hematology and Oncology, VA Southern Nevada Healthcare System, Las Vegas, USA

**Keywords:** hfe h63d mutation, skin blister, high ferritin, porphyria cutanea tarda, hemochromatosis

## Abstract

Hereditary hemochromatosis is an autosomal recessive condition with incomplete penetrance that is most commonly caused by a mutation in the HFE gene. Hereditary hemochromatosis can remain asymptomatic in some patients until triggered by certain events. Porphyria cutanea tarda is a condition that can lead to iron overload due to defective synthesis of heme and can cause the onset of adult-onset hereditary hemochromatosis. Herein, we present a case where a 77-year-old man presented with painful blisters on the sun-exposed areas of his hands and was diagnosed with porphyria cutanea tarda. Further testing for mutations in the HFE gene given elevated ferritin was performed and returned positive, which confirmed the diagnosis of adult-onset hereditary hemochromatosis. The patient received serial therapeutic phlebotomy for iron overload and adopted lifestyle modifications such as avoiding sun exposure of upper extremities. The patient’s blisters and laboratory iron panel parameters improved with continued phlebotomy. Therapeutic phlebotomy has been demonstrated to be an effective first-line therapy in patients with dual diagnosis. Our case highlights that cutaneous symptoms due to porphyria cutanea tarda may be the first presenting symptom in patients with underlying hemochromatosis.

## Introduction

Hereditary hemochromatosis (HH) is an autosomal recessive, adult-onset condition characterized by impaired iron metabolism that leads to excessive iron storage within the body [1,2]. There are several different mutations that can cause HH; the most commonly found mutation is that of the homeostatic iron regulator (HFE) gene, located on chromosome 6 [2]. The HFE gene encodes a protein that regulates the action between the transferrin receptor with transferrin. A defective HFE protein can result in excessive intracellular iron accumulation. HFE mutations can affect one in 400 individuals in America, and as high as one in 10 Americans of Northern European descent [1]. However, HH displays incomplete penetrance pattern, and a mutation alone is not indicative of development of hemochromatosis in life [3]. Other factors can be contributory as well, one of which is porphyria cutanea tarda.

Porphyria cutanea tarda (PCT) is a rare disease with a prevalence of one in 25,000 in the United States and can be familial or sporadic. Defective or inhibited uroporphyrinogen decarboxylase leads to faulty biosynthesis of heme, which results in accumulation of large amounts of porphyrins [4]. The proteins are then deposited in the body and cause the blistering condition on sun-exposed areas of the body. PCT can also be associated with hepatic iron overload; genetic studies have shown a 60-80% prevalence of HFE gene mutations in patients with PCT [2]. Herein, we present a case where a patient presenting with PCT was found to have mutations at the HFE gene and was diagnosed with adult-onset hereditary hemochromatosis.

## Case presentation

**Figure 1:**
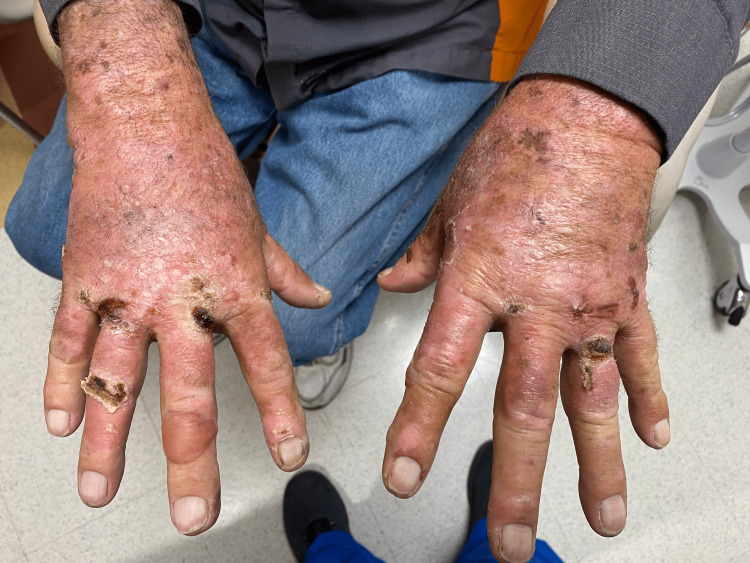
Persistent cutaneous blisters despite supportive skin care

A 77-year-old male presented to his primary care physician with complaints of blisters on the dorsal and ventral surfaces of both of his hands (Figure 1). The blisters were described as painful, dry, hyperpigmented lesions without drainage at different sizes and at different stages of healing. He was evaluated in the emergency department several months prior for similar symptoms and was treated with skin emollients and aspiration of the blister. His blistering condition did not improve despite the treatment, and he presented to the primary care clinic. His past medical history included unexplained polycythemia with a hemoglobin of 20 g/dL, type 2 diabetes mellitus, atrial fibrillation, hyperlipidemia, and hypothyroidism. The patient did not take anticoagulants as a home medication. Polycythemia had been noted in the past year, while his other chronic conditions had been present for several years. The patient was diagnosed with diabetes when he was at the age of 70 with current glycated hemoglobin (hemoglobin A1c) of 7.4; however, he did not have polycythemia until the past year, or blisters until several months prior.

Laboratory data were significant for elevated levels of transferrin saturation at 76%, serum iron at 246 mcg/dL, serum ferritin level at 477.9 ng/mL, and hemoglobin at 20 g/dL. Other labs including hematocrit, transferrin, and transferrin saturation index were within reference limits. Total serum porphyrins were also notably elevated at 199.5 mcg/L. A reflex 24-hour urine test was performed showing elevations in hepta, hexa, and penta-carboxyl porphyrins and uroporphyrin. Urine porphobilinogen was within normal limits. These findings supported a diagnosis of porphyria cutanea tarda.

However, given his elevated ferritin and iron saturation, genetic testing for hemochromatosis was performed. Hemochromatosis gene studies demonstrated heterozygous C282Y and H63D mutations at the HFE gene. Abdominal ultrasound revealed a mildly coarse and enlarged liver measuring 19.9 cm. Transient elastography demonstrated liver stiffness measurement at 5.1 kPa, which indicated no fibrosis, and controlled attenuation parameter at 272 dB/m, which suggested medium steatosis. Screening was unremarkable for other causes of chronic liver disease including but not limited to viral hepatitis through hepatitis serology, Wilson disease by negative ceruloplasmin, autoimmune hepatitis via a negative autoimmune workup, and alpha 1 antitrypsin deficiency via normal alpha 1 antitrypsin levels (Table 1). The patient was subsequently diagnosed with adult hereditary hemochromatosis. The patient denied hemochromatosis in his family history.

**Table 1:** Laboratory data of chronic liver disease workup in the patient IgM = Immunoglobulin M IgG = Immunoglobulin G

Disease	Testing	Results	Reference range
Viral Hepatitis	Hepatitis A antibody IgM	Non-reactive	Non-reactive
	Hepatitis B core antibody IgM	Non-reactive	Non-reactive
	Hepatitis B core antibody IgG	Non-reactive	Non-reactive
	Hepatitis B surface antigen	Non-reactive	Non-reactive
	Hepatitis C antibody	Non-reactive	Non-reactive
Wilson disease	Serum ceruloplasmin	28.5 mg/dL	19.0-39.0 mg/dL
	Neuropsychiatric symptoms	Absent	Absent
	Kayser Fleischerr Rings	Absent	Absent
Autoimmune hepatitis	Alanine aminotransferase	23 U/L	0-55 U/L
	Aspartate aminotransferase	27 U/L	5-34 U/L
	Antinuclear antibody	<1:80	<1:80
	Anti-smooth muscle antibody IgG	14	0-19 units
Alpha 1 Antitrypsin Deficiency	Alpha 1 antitrypsin	130 mg/dL	90.0-200.0 mg/dL

**Figure 2:**
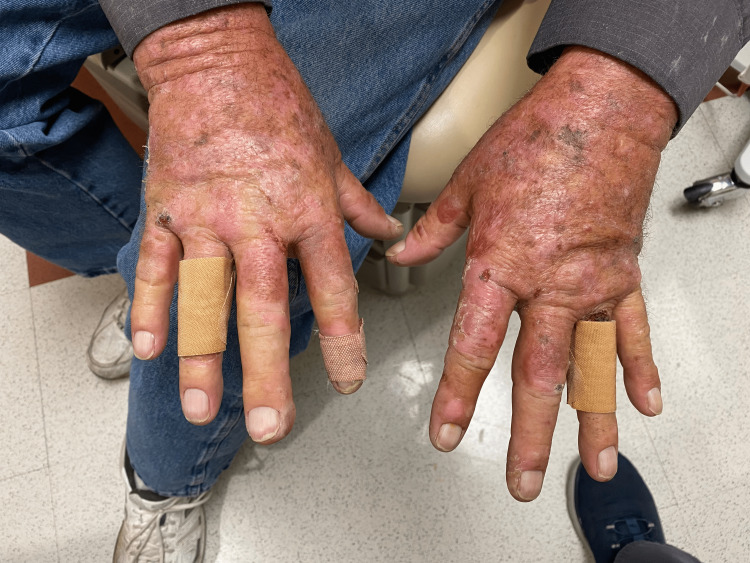
Cutaneous blisters in different stages of healing on the dorsal surface

**Figure 3:**
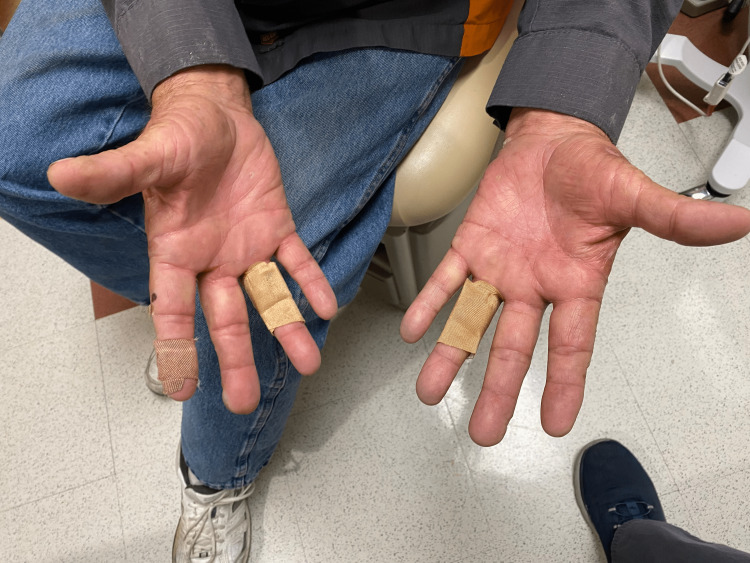
Cutaneous blisters in different stages of healing on the ventral surface

The patient was referred to oncology and was initiated on serial therapeutic phlebotomy given the state of iron overload. The patient was also recommended to avoid sun exposure, to use bacitracin, neomycin and polymyxin B antibiotic ointment, and to use doxycycline when the blistering worsened with explicit instructions to avoid further sun exposure when this does occur. Approximately six months after diagnosis and continued monthly phlebotomy, the patient had not experienced new blisters; the existing blisters were healing as well (Figure 2 and Figure 3). Serum ferritin had declined to the target level of 50-100 ng/mL. Serum iron and iron saturation were within reference limits at 123 mcg/dL and 38%, respectively (Table 2).

**Table 2:** Change in iron panel six months after continued phlebotomy

Test	At presentation	6 months Post Phlebotomy
Ferritin	477.9 ng/mL	50 ng/ml
Serum iron	246 mcg/dL	123 mcg/dL
Transferrin Saturation	76%	38%

## Discussion

Our patient demonstrates an unusual case where hemochromatosis manifested as porphyria cutanea tarda. Our patient was found to have a heterozygous HFE mutation at C282Y and H63D. A mutation in C282Y is the most common mutation in patients with hereditary hemochromatosis while mutations in H63D are more rare [5]. Prevalence of the different HFE gene mutations in patients with porphyria cutanea tarda were estimated at 23% heterozygous and 19% homozygous for C282Y and 23% heterozygous and 8% homozygous for H63D. Excess iron stores in the body because of the HFE mutation, combined with other lifestyle factors, can result in increasing amounts of heme production. Consequently, large amounts of porphyrins are produced and accumulate within the body which can lead to cutaneous blisters and hepatic injury [6]. Therefore, it was concluded that mild mutations in these genes were prevalent in patients with PCT and any insult that led to iron overload would result in the development of symptoms.

Similar findings were reported in a case series by Mehrany et al. [3]. Mutations in C282Y and H63D may indicate underlying HH and predispose patients to developing PCT [3]. However, given its incomplete penetrance and variable clinical expression, confirmation of the mutation does not predict age at onset of symptoms or disease severity [3]. However, there have been reports in the literature that a dual diagnosis of both HH and PCT may cause pediatric onset of these typically adult-onset conditions [6].

## Conclusions

Since HH can present as non-specific symptoms, PCT may be the first manifestation of HH that brings a patient to a healthcare professional. Therefore, when a blistering condition is seen and porphyria is suspected, genetic testing for HFE mutations may be considered to screen for underlying HH, which is treatable but may lead to cirrhosis if not discovered in time. Treatment focuses on controlling total body iron via therapeutic phlebotomy and avoiding any risk factors that may promote iron uptake. Phlebotomy causes mobilization of iron and porphyrin and stimulates the activity of the enzyme uroporphyrinogen decarboxylase. Therapeutic phlebotomy may be the most effective first-line therapy in patients with dual diagnosis. Hydroxychloroquine may also be considered for the treatment of the cutaneous blisters by solubilizing porphyrin molecules for urinary excretion.
